# The risk of low energy availability among athlete females in Saudi Arabia: a cross-sectional study

**DOI:** 10.3389/fpubh.2024.1411724

**Published:** 2024-05-30

**Authors:** Mai A. Khatib, Elham A. Aljaaly, Maryam S. Hafiz, Alkhuzama Alamri, Wejdan Alzahrani

**Affiliations:** ^1^Department of Clinical Nutrition, Faculty of Applied Medical Sciences, King Abdulaziz University, Jeddah, Saudi Arabia; ^2^Food, Nutrition, and Lifestyle Unit, King Fahd Medical Research Center, King Abdulaziz University, Jeddah, Saudi Arabia; ^3^Obesity Unit, King Abdulaziz University Hospital, King Abdulaziz University, Jeddah, Saudi Arabia; ^4^Adult Podiatric Adolescent Obesity Clinic, MNT Unit, King Abdulaziz University Hospital, King Abdulaziz University, Jeddah, Saudi Arabia

**Keywords:** exercise addiction, eating disorders, female athlete triad, amenorrhea, relative energy deficiency in sport

## Abstract

**Introduction:**

Low energy availability (LEA) is a state of inadequate energy reserves that results from a negative energy balance. This condition can lead to severe health risks such as amenorrhea and osteoporosis. Various causes for LEA, such as eating disorders and exercise addiction, have been reported in the literature. However, data in Saudi Arabia are lacking. This cross-sectional study measures the prevalence of LEA, eating disorders, and exercise addiction among adult females in Saudi Arabia and identifies possible associated risk factors.

**Methods:**

The sample comprised 119 female athletes who filled out an online survey adapted from the LEA in Females Questionnaire, the Eating Disorder Examination Questionnaire, and the Exercise Addiction Inventory.

**Results:**

Participants showed a high prevalence of LEA (66.4%), eating disorder (33.6%), and exercise addiction (10.1%), confirming the association between normal weight and LEA in females living in Saudi Arabia (*p* < 0.00).

**Discussion and conclusion:**

With an increasing number of females in the country interested in following a healthy lifestyle, there is a need to raise the awareness of the population on the issues of LEA, eating disorders, and exercise addiction and their effects on the body by developing educational programs about energy intake and healthy physical activity routines.

## Introduction

Energy availability is the dietary energy left over to support physiological functions after deducting energy expenditure from exercise (1). Inadequate intake and/or excessive expenditure of energy may lead to a state known as low energy availability (LEA), where the body's ability to support optimal physiological function is compromised (2). Endurance athletes typically exhibit LEA resulting from altered dietary habits and/or high energy expenditure brought on by body dissatisfaction, the notion that becoming lighter will enhance performance and social pressure to maintain a particular appearance. Additionally, athletes may feel pressure from their teammates, coach, or social media (2). It has been suggested that energy savings may serve as a helpful indicator of LEA. In competitive female cyclists, poor aerobic performance and relative energy shortage were both linked to female riders with ratios of measured resting energy expenditure to predicted resting energy expenditure (mREE/pREE) below 0.9, which are associated with LEA (3). Low energy availability in female endurance athletes who do not have disordered eating behavior is common, and studies have stated that it is more common among athletes engaging in weight-sensitive sports when in comparison with sedentary controls (4). Low Energy Availability in Females (LEAF), particularly, leads to undesirable conditions such as poor resting energy expenditure and disruption of a variety of hormonal, metabolic, and functional features; although both genders can develop LEA, which lowers endurance, raises the risk of injury, and depletes glycogen stores (5). As a result, the body attempts to conserve energy, through metabolic changes that maintain homeostasis by decreasing energy expenditure (3). Reduced macronutrient intake in athletes may result in a decline in their physiological capacity for bone production, maintenance of muscle mass, repair of damaged tissue, and recovery after injury (6). Furthermore, during periods of intense exercise training, glycogen stores may not be adequately restored especially when carbohydrate consumption prior the exercise was not insufficient. Moreover, protein needs might also increase since the protein reserves could be utilized as a substitute source of energy. Additionally, micronutrients are necessary for the growth of bones and muscles, the replacement of erythrocytes, and the availability of cofactors for the control of metabolic reactions that produce energy (6). For this reason, a well-balanced and diverse diet that provides all macronutrient needs as well as vitamins, minerals, and sufficient energy should be maintained (7). Insufficient availability of energy results in reduction of energy expenditure by stopping bodily functions, including menstrual periods, hence the main concern will be survival (7). Functional hypothalamic amenorrhea is known as the menstruation absence caused by supressed axis of hypothalamus to ovaries with absence of organic or anatomical reasons (8). It is potentially reversible and frequently manifests itself in conditions of stress, rapid weight loss, and overexertion (8). Usually, secondary amenorrhea, or previously regular menses that cease for at least 3 months or menses already irregular that cease for at least 6 months, calls for evaluation (9). Psychological variables in eating patterns, as in disordered eating, may cause LEA, although LEA in turn can cause severe psychological suffering (10). The two factors, amenorrhea and eating disorders, that come with LEA are also important components called female athlete triad (FAT) (10). The FAT, which includes amenorrhea, osteoporosis, and an eating disorder, was initially identified in 1992 (11). Currently, it has been proposed that the FAT can be defined as the existence of one or more elements in females participating in sports (11). Screening tools for early detection of FAT/LEA symptoms are crucial to protect young athletes from long-term harm brought on by the development potential risks connected to FAT/LEA (12). Several quantitative measurements can be used to test for LEAF, including body compositional analysis, bone mineral density, basal energy expenditure, and day-to-day dietary recall (12). Moreover, a qualitative instrument can be utilized such as the Low Energy Availability in Females Questionnaire (LEAF-Q) (12). It was once thought that the triad only affected professional athletes, but we now know that it can even affect women who are not athletes (11).

Moreover, early detection of factors associated with LEA can aid in preventing deterioration in performance and health and in planning protection and proper nutritional intervention programs (10). Specifically, disordered eating behavior was commonly shown among weight-sensitive athletes and considered a significant risk factor for developing LEA (2, 13), although LEA among female endurance athletes without disordered eating was also shown to be common (14). The gold standard for detecting the behavioral symptoms of disordered eating is Eating Disorder Examination Questionnaire (EDE-Q) 6.0 (15, 16).

Physical activity, universally recognized as a healthy habit, has the potential to develop into an unhealthy preoccupation, referred to as exercise addiction (17). The primary addiction to exercise is characterized by exercise addiction that does not accompany disordered eating; while secondary, which can develop in conjunction with eating disorders or because of them (18). Low energy availability can result from either type, but it is still unknown how exercise addiction, separate from disordered eating, contributes to the development of LEA (13, 19). Also, exercise addiction not only runs the risk of causing considerable bodily harm, but those who struggle with this addiction also prioritize exercise over their relationships with family and friends, their health, and their careers (17). Assessment for the risk of exercise addiction is often neglected by health care professionals (17). However, it can be screened for using the Exercise Addiction Inventory Questionnaire (EAI-Q), which is a quick and easy. Based on the available literature on LEAF, more research is required to investigate the link connecting disordered eating behavior to exercise addiction with LEAF, especially in Saudi Arabia that has seen an increasing numbers of females of reproductive age participating in sports. Therefore, the main purpose of this study was to measure the prevalence of LEAF, exercise addiction, and eating disorders among females in Saudi Arabia and to identify the association between LEAF in female athletes with exercise addiction, body mass index, and eating disorders. This will help to understand the magnitude of this problem in the society and aid the Ministry of Health in developing proper approaches and educational programs about appropriate energy intake in addition to helping to understand the effect of healthy food intake and good exercise at the population level.

## Materials and methodology

### Participants

The sample size was calculated using Epi Info 7.2.4.0 (CDC, Atlanta). Based on the female population in Saudi Arabia in 2022, with an 80% confidence level, 50% expected frequency, and a 5% margin of error, the estimated sample size required for this study was 164 participants. Recruitment took place through the snowball sampling method. Invitations were sent electronically via social media outlets. We included female athletes [who perform 5 h per week or more (20)] from any region of the country, age 18–50 years old, who had no medical conditions that could cause LEA. We excluded women who are active yet perform < 5 h per week, females of menopausal period, females with history of any chronic disease and/or who have taken hormonal replacement therapy over the past year, women taking medication that affects bone mineral density, and pregnant or breastfeeding females prior or at during the study.

### Measures

The study was cross-sectional that was conducted from September 2022 until June 2023, female athletes filled out an online questionnaire adapted from three previously validated and published questionnaires (14, 21, 22) concerned with energy availability in females, exercise addiction, and the evaluation of disordered eating behavior in physically active females. The questionnaire also collected demographic data including age, educational level, profession, height, weight, BMI, medical background, use of medications, and smoking (Table 1).

**Table 1:** Participant sociodemographic and lifestyle characteristics and Low Energy Availability in Females (LEAF) scores (*N* = 119).

**Factor**		**All**	**LEAF-Q score < 8**	**LEAF-Q score ≥8**	***p-*value**
		***N*** = **119**	***n*** = **40**	***n*** = **79**	
Age		21.92 ± 4.54	22.78 ± 5.12	21.49 ± 4.18	0.17
Education (%)	Below secondary school	4.2	7.5	2.5	0.08
	Secondary school	29.4	25	31.6	
	Bachelor	60.5	55	63.3	
	Postgraduate	5.9	12.5	2.5	
Employment (%)	Full time	9.2	10	8.9	0.65
	Part time	9.2	12.5	7.6	
	Unemployed	81.5	77.5	83.5	
Do you have any medical background? (%)	Yes	26.9	32.5	24.1	0.64
	No	73.1	67.5	75.9	
BMI	Underweight	10.9	10	11.4	0.05^*^
	Normal	63.9	50	70.9	
	Overweight	17.6	30	11.4	
	Obese	7.6	10	6.3	
Height (cm)		159 ± 6.13	161.08 ± 5.55	158 ± 6.18	0.007^**^
Weight (kg)		57.47 ± 10.74	60.92 ±10.21	55.73 ± 10.65	0.01^*^
BMI		22.68 ± 3.82	23.49 ± 3.77	22.28 ±3.80	0.1
Exercise (hours/week)		5.14 ± 2.39	5.05 ± 2.57	5.19 ± 2.31	0.76
Do you smoke? (%)	Yes	4.2	2.5	5.1	0.51
	No	95.8	97.5	94.9	
Do you use any medication (excluding oral contraceptives)? (%)	Yes	7.6	5.0	8.9	0.45
	No	92.4	95.0	91.1	
LEAF score		9.57 ± 3.95	5.47 ± 1.44	11.65 ± 3.09	< 0.001^***^
EDE global score		2.14 ± 1.21	2.18 ± 1.23	2.11 ± 1.21	0.76
EAI score		18.77 ± 3.94	18.07 ± 3.77	19.12 ± 4.01	0.16

Data are represented as mean and standard deviation for continuous data and percentage for nominal data. LEAF-Q score ≥ 8 indicates at risk for LEA. LEAF is the dependent variable (low risk and at risk). Wilcoxon rank-sum and chi-square tests were used.

BMI, body mass index; LEAF-Q, Low Energy Availability in Females Questionnaire; EDE, Eating Disorder Examination; EAI, Exercise Addiction Inventory.

^*^p ≤ 0.05.

^**^p ≤ 0.01.

^***^p ≤ 0.001.

Prior to the study, all four sections of the tool were reviewed, modified, and refined by an expert panel of seven clinical and sports nutrition professionals. The expert panel also observed and approved the translation of the tool into Arabic. To confirm the questionnaire's validity and reliability, it was pilot tested on seven students, who were similar in age to the study population, following the same inclusion criteria, results of which are included in the analysis. The tool was shared with participants all over the country electronically through social media platforms [WhatsApp, X (formerly Twitter), and Instagram] using a poster with the survey barcode.

### Ethical approval

The ethical approval for the study was obtained from the Unit of Biomedical Ethics at King Abdulaziz University (Reference number: FAMS-EC2023-01). Strict confidentiality was upheld for the sample and the data collected. Data were de-identified during evaluation, analysis, and any publication. Electronically signed consent was obtained from all participants prior enrolment in the study and answering questions.

### Statistical analysis

Statistical analysis was carried out using IBM SPSS Statistics (Version 23.0) with double -tailed significance level set at *p* ≤ 0.05. The data was inspected and verified as non-normally distributed using one sample Kolmogorov–Smirnov test (*p* < 0.01), thus the Wilcoxon rank-sum test and score and Mann-Whitney *U*-test were used. The results are presented as median and interquartile range (Q1 at 25% and Q3 at 75%). We used chi-square test to investigate possible differences among categorical variables in two independent groups. Finally, we used logistic regression to identify potential risk factors for LEA, defined as a LEAF-Q score ≥ or < 8 as the dependent variable. Odds ratios and confidence intervals were used to investigate possible associations among logistic regression model. Variables were expressed as numbers and percentages. To reinforce clear and thorough reporting, the study followed the STROBE checklist (23).

## Results

### Sociodemographic factors

The total number of responses to the questionnaire received was 184. However, only 119 responses were considered in the analysis after excluding responses with no training status or respondents who did not fulfill the criterion used to define athletes (exercising for five or more hours per week) (20), which is crucial when using the LEAF questionnaire as it was validated for use with female athletes (12, 14) (see Table 1 for sociodemographic data). Average participant age was 21.92 ± 4.54 years. Average body mass index (BMI) was 22.68 ± 3.82 kg/m^2^ (63.90% had a normal BMI). Only five participants were smokers.

### Energy availability risk score

Seventy-nine participants scored 8 or higher on the LEAF-Q (66.4%), qualifying them to be at risk for LEA, and the difference was statistically significant from the number of participants at low risk (40 participants, 33.6%), *p* < 0.001. Forty participants scored 2.5 or higher on the EDE-Q, qualifying them to be statistically significant at risk for eating disorders (33.6%; *p* < 0.001). Twelve participants had statistically significant risk for exercise addiction with a score of 24 or higher on the EAI-Q, *p* < 0.001. Among the participants with LEA (*n* = 79), four (3.3%) were classified as having primary exercise addiction, 6 (5.0%) had secondary exercise addiction, and 20 (16.8%) had disordered eating without exercise addiction.

We found no significant relationship between LEA and eating disorders (*p* > 0.05), although 40% of subjects that were at greater risk of LEA also scored 2.5 or higher on the EDE-Q (Table 2). No relationship was found between LEA and exercise addiction either (*p* > 0.05), although 12.7% of subjects that were at greater risk of LEA also scored 24 or higher on the EAI-Q (Table 3). The results revealed a significant association between having a BMI in the normal or underweight range and being at risk for LEA (*p* < 0.05), as 11.2% of the participants who were underweight according to BMI and 63.2% of participants with normal weight were at greater risk for LEA (LEAF-Q score ≥ 8; Table 4). Logistic regression analysis has revealed that low BMIs were at greater risk of LEA (OR = 0.29; *p* ≤ 0.01), meaning that BMI can be considered a contributing factor for LEAF (Table 5). There was no statistically significant association between LEA and any of the other variables (Table 5).

**Table 2:** Relationship between eating disorders and Low Energy Availability in Females score.

**EDE-Q**	**LEAF-Q**				***p-*value**
	<**8**		≥**8**		
	**No**.	**%**	**No**.	**%**	
< 2.5 (%)	26	65.0%	53	67.1%	0.82
≥2.5 (%)	14	35.0%	26	32.9%	
Total	40	100.0%	79	100.0%	

EDE-Q scores ≥ 2.5 indicate individuals at risk for eating disorders. LEAF-Q scores ≥ 8 indicate individuals at risk for LEA. LEAF score is the dependent variable (low risk and at risk).

Chi-square test was used.

EDE-Q, Eating Disorders Evaluation Questionnaire; LEAF-Q, Low Energy Availability in Females Questionnaire.

**Table 3:** Relationship between exercise addiction and Low Energy Availability in Females score.

**EAI score**	**LEAF-Q score**				***p*-value**
	<**8**		≥**8**		
	**No**.	**%**	**No**.	**%**	
< 24	38	95.0%	69	87.3%	0.19
≥24	2	5.0%	10	12.7%	
Total	40	100.0%	79	100.0%	

EAI scores ≥ 24 indicate that the individual is at risk of exercise addiction. LEAF-Q scores ≥ 8 indicate individuals at risk of LEA. LEAF score is the dependent variable (low risk and at risk). Chi-square test was used.

EAI, Exercise Addiction Inventory; LEAF-Q, Low Energy Availability in Females Questionnaire.

**Table 4:** Relationship between BMI and Low Energy Availability in Females score.

		**LEAF group**				**Total**		
		**No**		**Yes**				
		**No**.	**%**	**No**.	**%**	**No**.	**%**	***p*** **-value**
BMI level	Underweight	4	10.0%	9	11.4%	13	10.9%	0.05^*^
	Normal	20	50.0%	56	70.9%	76	63.9%	
	Overweight	12	30.0%	9	11.4%	21	17.6%	
	Obese	4	10.0%	5	6.3%	9	7.6%	
Total	40	100.0%	79	100.0%	119	100.0%		

BMI < 18.5 indicates underweight; between 18.5 and 25 indicates normal weight; between 25 and 30 indicates overweight; >30 indicates obesity. LEAF-Q scores ≥ 8 indicate individuals at risk for LEA. LEAF score is the dependent variable (low risk and at risk).

BMI, body mass index; LEAF-Q, Low Energy Availability in Females Questionnaire.

Chi-square test was used.

^*^p ≤ 0.05.

**Table 5:** Risk factors for low energy availability.

		**OR**	**95% CI**		***p*-value**
			**Lower**	**Upper**	
Independent variable	BMI	0.295	0.124	0.704	0.006^*^
	EAI	2.75	0.573	13.222	0.20
	EDE	0.91	0.409	2.031	0.82

The dependent variable is low energy availability in females score. A binary logistic test was conducted.

OR, odds ratio; CI, confidence interval; BMI, body mass index; EAI, Exercise Addiction Inventory; EDE, Eating Disorder Examination.

^*^p ≤ 0.01.

## Discussion

The main objective of our study was to measure the prevalence of LEA among active adult females in Saudi Arabia and to identify the contributing factors associated with LEAF. The study found a significant correlation between BMI and LEA; lower BMI status was more prevalent with participants at greater risk for LEA. Our study showed that 66.4% of female athletes were at high risk for LEA, which is similar to 65 and 62.2% cases of LEA in female athletes reported by Fahrenholtz et al. (4) and Melin et al. (14), respectively. However, a lower percentage (31%) was reported by Carr et al. (24), and a higher percentage (79.5%) was reported by Jesus et al. (24, 25). These discrepancies could be related to the different types of sports practiced and the level of activity (26). Despite the reasons associated with LEA, the high prevalence indicates vulnerability of the studied group of females to LEA, which can, consequently, lead to a rise of symptoms related to FAT and relative energy deficiency in sport (RED-S) syndromes (19).

For the majority of female athletes, LEA results unintentionally from appetite suppression post exercise (19, 27), low energy diets (28), An insufficient understanding of ideal sports nutrition (16, 29–31) along with impacts of LEA (32–35), or a busy lifestyle with inadequate time or access to food (29, 36, 37). On the other hand, insufficient balance between caloric intake and expenditure resulting in LEA may lead intentionally in pursuit of optimizing body mass and composition, to avoid gaining weight while downtime, or as a result of eating disorders or exercise dependence and/or addiction (36, 38), although, as previously mentioned, female endurance athletes can have LEA without disordered eating (39). Nevertheless, the present study showed prevalence rates of 32.9 and 12.7% for eating disorders and EA, respectively, among female athletes at high risk for LEA. On the other hand, data analysis revealed no significant association between eating disorders and LEA or between exercise addiction and LEA. This comes contrary to what is published in the literature. Fahrenholtz et al. (4) demonstrated that participants at greater risk for LEA also had high scores for both disordered eating and exercise addiction when compared with participants at low risk (4). This can explain the high prevalence of LEA (62.2%) in their study, since availability of energy results from energy intake and expenditure, and, thus, both disordered eating and exercise addiction should be considered when examining for LEA (4). Collectively, our results suggest a high prevalence of eating disorders and exercise addiction in women, which is concerning.

There are primary and secondary forms of exercise dependence. When it is caused by disordered eating or is associated with it, the condition is secondary (40). However, if it occurs without accompanying symptoms of disordered eating, it is primary exercise dependence (41–44). In the latter, the person exercises continuously for the sole purpose of psychological gratification that results from the exercise itself no other pathologies (40). The current findings show that among the athletes with LEA (*n* = 79, 66.4%), primary exercise addiction was found in 3.3%, 5.0% had secondary exercise addiction, and 16.8% had disordered eating without exercise addiction. Disordered eating is known as a reason that can cause LEA (19, 45); however, how exercise dependence causes LEA in the absence of disordered eating has not yet been explored. Preliminary studies have pointed to an increase in biochemical markers that indicate LEA (13, 46). However, these results are limited. It is possible that reduced availability of energy may contribute to the eating disorders in secondary exercise dependence. It is crucial, therefore, when developing interventions for athletes with RED-S syndrome to examine both dietary and exercise patterns.

In the present study, a rough measure of BMI was used with the intention to generally investigate the association between BMI and LEA among the population of female athletes. The current findings showed lower BMIs in athletes at greater risk for LEA comparing with athletes at low risk. Moreover, using regression analysis, LEA was found to be influenced by BMI. This could be logically expected, as athletes generally perceive excess fat as a major limiting factor in sport performance and that a higher skeletal muscle mass promotes strength and power (47). Thus, athletes might intentionally decrease their intake in order to reach the desired shape and/or optimal body composition (48). One can speculate from this point of view that LEA is not exclusive to a certain weight group and that it can be suspected even in people of normal weight. This highlights how important it is to promote healthy dietary habits and a positive body image among female athletes, since the current findings showed that most of the athletes at risk for LEA presented a healthy BMI. Although the current outcomes are in line with those of Fahrenholtz et al. and Christo et al. (4, 49), other reports disagree with them (14, 28). This conflict can only suggest that BMI cannot be used alone when screening for LEA, given the potential metabolic compensatory mechanisms and that other measurements of body composition should be used to carefully identify malnutrition and examine athletic health and performance (50–52). Further research is recommended to elucidate this relationship.

The novelty in this study is the identified prevalence of LEA, eating disorders, along with exercise addiction among females residing in the various regions of Saudi Arabia. The outcomes of the current research emphasize the importance of the topic and reinforce the need for larger future studies that allow for the detection of comparable differences among the different regions of the country. Due to the cross-sectional design of the study, it was not possible to identify the correlation's direction and, hence, causality. Moreover, hence the data collection involved self-reporting, it might be prone to response bias, denial, and erroneous reporting in studies. Overestimation or underestimation of anthropometric data and food intake was also an issue. Future studies should include measuring the nutritional knowledge of the participated females and categorizing the participated females according to the type of sport practiced during analysis and risk association, since athletes in weight categories of aesthetic sports manipulate their weight when compared to other type of sports. In conclusion, this study showed a high prevalence of LEA (66.4%), eating disorders (33.6%), and exercise addiction (10.1%) and confirmed the association between normal weight and LEA in females living in Saudi Arabia. The high percentage of the affected population elicits the need to raise awareness among females, especially with the increasing trends of practicing sports and following a healthy lifestyle in the country. Further, larger studies should follow to assess the causative relationship between LEA and eating disorders, exercise addiction, and BMI in both genders. The results of the current study should be used as rough estimates for future studies comparing prevalence rates with other Gulf, and neighboring, countries. They will also help the Ministry of Health in developing proper educational programs and approaches for appropriate energy intake as well as understanding the effect of healthy food intake and good exercise at the population level.

## Data availability statement

The raw data supporting the conclusions of this article will be made available by the authors, without undue reservation.

## Author contributions

MK: Conceptualization, Formal analysis, Resources, Supervision, Writing – original draft, Writing – review & editing. EA: Data curation, Investigation, Methodology, Supervision, Visualization, Writing – review & editing. MH: Methodology, Project administration, Supervision, Visualization, Writing – review & editing. AA: Investigation, Methodology, Writing – original draft, Writing – review & editing. WA: Investigation, Methodology, Writing – original draft, Writing – review & editing.
